# Inter-reader agreement and additive benefit of SPECT or SPECT/CT modality with [^99m^Tc]Tc-pertechnetate scintigraphy imaging for the assessment of thyroid nodules in a tertiary care center

**DOI:** 10.1186/s41824-025-00264-6

**Published:** 2025-08-18

**Authors:** Emil Novruzov, Dominik Schmitt, Katalin Mattes-György, Julian Kuhlmann, Mareike Muchalla, Matthias Schott, Christina Antke, Frederik L. Giesel, Eduards Mamlins

**Affiliations:** 1 Department of Nuclear Medicine, Medical Faculty and University Hospital Duesseldorf, Heinrich-Heine-University Duesseldorf, Moorenstrasse 5, 40225 Düsseldorf, Germany; 2 Department of Endocrinologiy, Medical Faculty, University Hospital Dusseldorf, Heinrich Heine University of Duesseldorf, Duesseldorf, Germany

**Keywords:** SPECT(-CT) imaging, Thyroid nodule, Goiter, [^99m^Tc]Tc-pertechnetate scintigraphy, Planar image, ACR TI-RADS

## Abstract

**Aim/introduction:**

In central Europe, up to 76% of population exhibit thyroid nodules (TN). The combination of ultrasound findings and thyroid scan imaging are supposed to ensure a cost-effective and reliable prediction of malignancy risk. However, real-world impact of ultrasound findings, i.e. TI-RADS, has raised concerns due to its highly limited reliability and reproducibility. The recent advancements and increasing access of SPECT/CT scanners might contribute to thyroid management. This study aimed to elucidate additive value of SPECT(-CT) imaging in initial evaluation of complex TN cases, particularly on multinodular goiter and difficult-to-characterize solitary nodules.

**Materials and methods:**

A total of 61 patients (19 males and 42 females with a mean age of 56 (± 17)) with incidental TN were retrospectively enrolled in this study between November 2022 and July 2023, who underwent both conventional planar thyroid scan and SPECT(-CT) imaging. Three readers with varying experience level selected a target nodule within thyroid gland and evaluated its functionality, ACR TI-RADS categorization and additive benefit of SPECT(-/CT) imaging. Kappa-value for inter-reader agreement (IRA) was interpreted as follows: < 0.01–0.20, slight agreement; 0.21–0.40, fair agreement; 0.41–0.60, moderate agreement; 0.61–0.80, substantial agreement; and 0.81–1.00; almost perfect agreement.

**Results:**

Of the target TNs, 33 (54%) were located in the left thyroid lobe, 5 (8%) in the isthmus and the remaining target TNs (38%) in the right lobe. The median target TN size was 20 mm (10–59). The target nodule match rate among readers was almost perfect (k-value 0.81; 95% CI: 0.72–0.90). The IRA regarding ACR TI-RADS was moderate with k-value of 0.47 (95% CI: 0.40–0.54). The IRA regarding patient referral was substantial among readers with k-value of 0.60, while k-value was even higher at pairwise comparison of experienced readers (k-value: 0.64). IRA regarding the target TN functionality among readers was good with k-value of 0.68 (95%CI: 0.59–0.77). IRA involving the less experienced reader revealed only slightly lower reliability scores, i.e. a k-value of 0.61. The correlation with ground truth results revealed comparable results among experienced and less-experienced readers.

**Conclusion:**

Increasing clinical experience of physicians appeared to have a direct correlation with a higher referral rate of SPECT(-CT) imaging, although the additive clinical benefit was only evident for a minority of patients. Hence, SPECT(-CT) imaging might be more suitable for tertiary care centers, as its additive benefit at primary care level would have only negligible effect for patients.

**Supplementary Information:**

The online version contains supplementary material available at 10.1186/s41824-025-00264-6.

## Introduction

Thyroid nodules (TN) are prevalent especially in regions with suboptimal iodine supply, e.g. central Europe. A German survey by Guth et al. reported a TN detection rate of up to 76% in healthy adults by a high-resolution ultrasonography examination. Mostly, these nodules are asymptomatic and only incidentally found in the underlying goiter. Owing to improved dietary iodine supply in recent decades, goiter in regular clinical care is nowadays only determined by ultrasonography in contrast to overwhelming clinical appearances in the past (Guth et al. 2009; Fiore et al. 2014). Given the fact that the majority of TN in regions with iodine deficiency are benign, picking out the high-risk nodule has been a challenge which would spare patients from overdiagnosis and -treatment as well as healthcare systems from an unnecessary burden (Low et al. 2022).

**Fig. 1:**
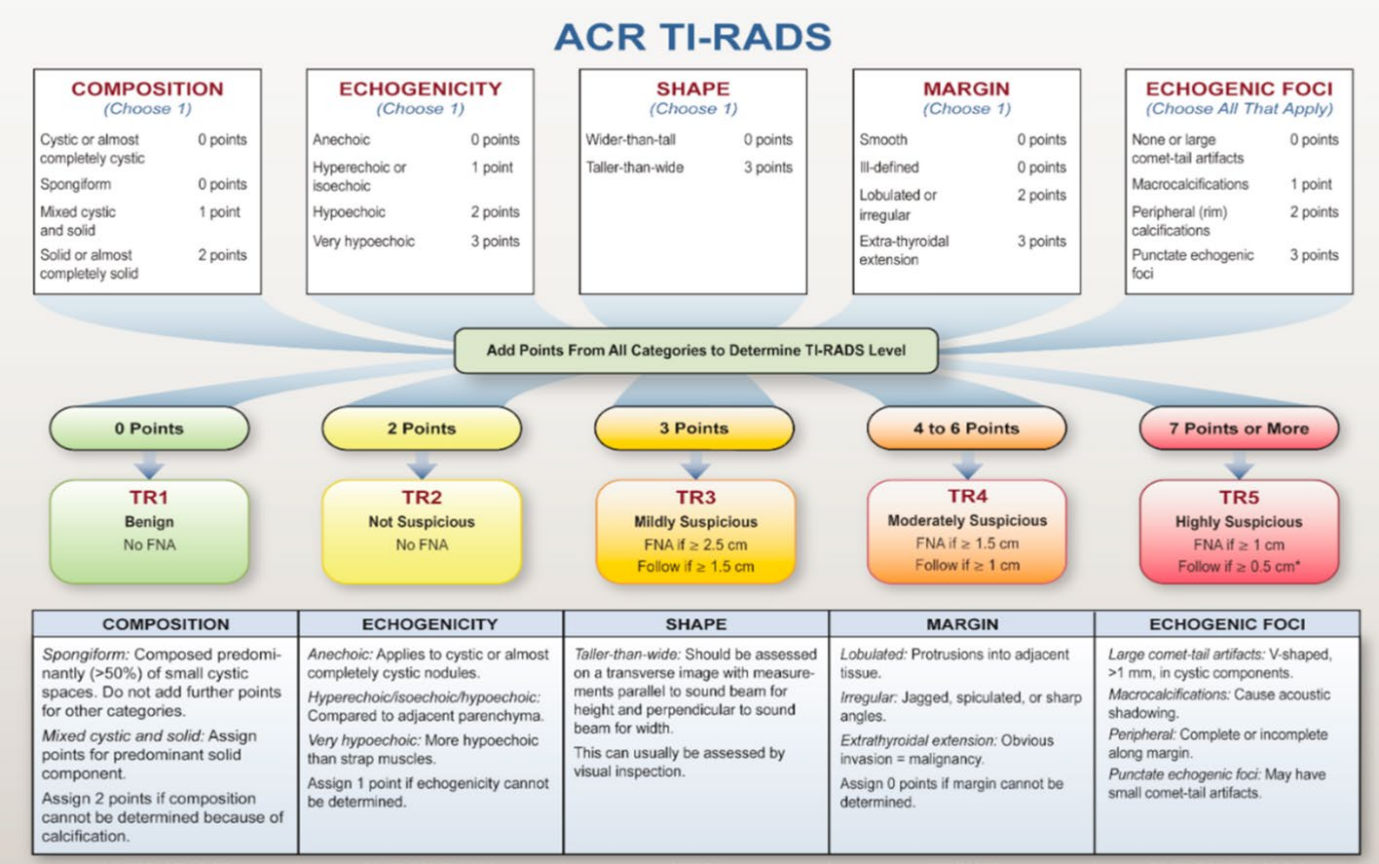
Depiction of ACR thyroid imaging, reporting and data system (TI-RADS) categories, adapted from Tessler et al. (2017)

Currently, ultrasonography examination represents the backbone of initial TN evaluation in regular clinical care. Thus, several societies introduced diagnostic frameworks deploying sonomorphological criteria in attempt to rule in TNs with features that, according to current understanding, correlate with an increased likelihood of malignancy. This approach is supposed to guide the clinicians more efficiently to stratify the patients and draw clinical conclusions such as reassurance or regular follow-ups, fine-needle-aspiration biopsy or surgical resection combined with histological validation and, eventually, spare patients from unnecessary biopsies or surgical interventions. These schemes are based upon the so-called TI-RADS framework and employ various sonomorphological criteria for a better prediction of malignancy risk. For instance, the American College of Radiology Thyroid Image Reporting and Data System (ACR TI-RADS) deploys 5 sonomorphological criteria involving composition, echogenicity, shape, margin, and echogenic foci to assign a TN to one of 5 categories: benign (TR1), not suspicious (TR2), mildly suspicious (TR3), moderately suspicious (TR4), or highly suspicious (TR5). A FNA is recommended only from TR3, whereas patients with TR1 or TR2 nodules are reassured (Fig. 1***)*** (Tessler et al. 2017).

In comparison to other guidelines based upon TI-RADS framework, ACR TI-RADS was reported to have the most favorable negative predictive value of 99.7% by the research group of Topcuoglu et al. (Topcuoglu et al. 2025) However, according to Wilkinson et al., real-world impact of ACR TI-RADS was highly questionable regarding the accurate prediction of malignancy, of which in only TR5 slightly increased malignancy rate was observed (Wilkinson et al. 2023). Moreover, the reliability and reproducibility of those guidelines have raised concerns (Alfuraih et al. 2024; Borges et al. 2023; Piticchio et al. 2024). Therefore, combination of [^99m^Tc] O_4_^−^(Pertechnetate) scintigraphy (thyroid scan) and ultrasonography is crucial in regions with a high TN prevalence, as unnecessary and high-risk invasive procedures could be avoided such as in hyperfunctioning TN. A retrospective study with a cohort size of 582 by Schenke et al. demonstrated that a substantial rate of patients with hyperfunctioning nodules could avoid invasive procedures only owing to the prior conduct of thyroid scan, as ultrasonography examinations had revealed malignancy features (Schenke et al. 2019). As a response to this phenomenon, guidelines of the German Society of Nuclear Medicine and German Association of Endocrine Surgeons recommend supplementary thyroid scan for TN larger than 1 cm regardless the TSH (thyroid-stimulating hormone) level which was also considered by the joint EANM practice guideline/SNMMI procedure standard for RAIU and thyroid scintigraphy (Dietlein, et al. 2022; Dralle et al. 2013; Giovanella et al. 2019). The current experience confirms the added value of this approach regarding a more accurate prediction of malignancy risk particularly in complex cases like multinodular goiter (Sollmann et al. 2024).

Moreover, a precise characterization of TNs on a multinodular goiter or a dorsal or substernal located singular nodule in goiter poses often challenges in regular clinical care. The guidelines recommend further imaging via single photon emission computed tomography (SPECT)/computed tomography (CT) (SPECT(-CT)) in the case of substernal or ectopic thyroid (Giovanella et al. 2019). The emergence of the combination of functional and anatomical map of the examined organs by SPECT(-CT) offers great potential regarding the utility of molecular imaging in patient management by providing improved reader confidence, reproducibility and more precise localization of tracer uptake in challenging regional thyroid locations. On the other hand, the implementation of SPECT(-CT) requires additional personnel and financial resources, particularly in primary and secondary nuclear medicine facilities ().

The guidelines underline the use of tomographical imaging rather for clarification of ectopic or substernal thyroid tissue, whereas the majority of pitfalls in regular clinical care might imply the accurate stratification of dorsally located TNs of interest on thyroid or a specific TN on a multinodular goiter. As a matter of fact, initial evaluation of TNs occur essentially in primary or secondary care facilities which need to operate efficiently and cost-effectively due to high patient turnover, reimbursement issues and relatively limited infrastructure. Given the recent advancement and better accessibility of hybrid imaging in thyroid management, the cost-effectivity of this imaging modality needs to be evaluated with respect to diagnostic work-up of TN, especially in complicated cases.

However, to our best knowledge, the literature data is very scarce regarding the additive benefit of SPECT or SPECT(-CT) imaging in diagnostic work-up of patients with multinodular goiter or uninodular goiter with a dorsal or substernal located nodules of interest. Hence, this study aims to elucidate the utility and additive value of tomographic and hybrid imaging in evaluation of complex TN cases on multinodular goiter and difficult-to-characterize solitary nodules.

## Material and method

A retrospective review of the local Picture Archiving and Communication System (PACS) of our institution of the time interval between November 2022 and July 2023 revealed 61 patients with incidental TN who underwent a conventional planar thyroid scan and additionally tomographical or hybrid imaging as an additive imaging in accordance with national and institutional guidelines. The routinely recorded clinical and laboratory parameters were also assessed such as thyroid function parameters such as TSH, free thyroxine, free triiodothyronine, and thyroid peroxidase-autoantibodies. An adequate assessment of thyroid scan mandates the knowledge of ultrasound examination providing adequate information about the morphology of the gland, especially location, size and sonographic features of thyroid nodules, hormonal status and a good image quality.

Thus, the inclusion criteria for this study were as follows:

- Fully accessible and eligible ultrasonographic examination on PACS
- Fully retrievable and complete cine video recording of ultrasound of thyroid gland on transaxial plane
- Fully accessible and comprehensible tomographic (SPECT technique) or hybrid imaging (SPECT/CT imaging)

Patients with insufficient image quality, incomplete thyroid gland or nodule visualization or absence of a cine clip covering were excluded.

This retrospective study was approved by the appropriate ethical committee and Institutional Review Board. All procedures performed in the clinical routine were in accordance with the ethical standards and the principles of the 1964 Declaration of Helsinki and its later amendments or comparable ethical standards.

### Acquisition of planar scintigram and SPECT(-CT) imaging

Starting 15 min after a mean 70 MBq [^99m^Tc]-pertechnetate injection, the planar thyroid images were acquired for 5–6 min over antero-posterior incidence of cervical region with the distance between the anterior head of the camera and the patients at 12 cm by a gamma camera. The planar acquisition was considered as the reference image for the thyroid uptake estimation. SPECT(-CT) imaging was acquired on three different dual-head gamma cameras with similar acquisition parameters. Non-enhanced CT was performed from the maxilla to the mid of corpus sterni. The detailed acquisition parameters of different scanners are summarized in Supplemental Table 1.

### Imaging interpretation

Thyroid scan images of pseudonymized patients were assessed by three readers using in-house standard workstations after retrieving data from PACS. Using Hermes software program (Hermia, Affinity 1.1.4; Hermia Medical Solutions, Stockholm, Sweden) for the complicated cases was also allowed. Two board-certified nuclear medicine physicians with a > 5-year and > 20-year clinical experience and a senior resident with an experience level of < 5 years reviewed all 61 patients independently. Only the actual clinical status including referral question, ultrasound and thyroid scan images alongside age and sex of the patients were unblinded to the readers.

**Fig. 2:**
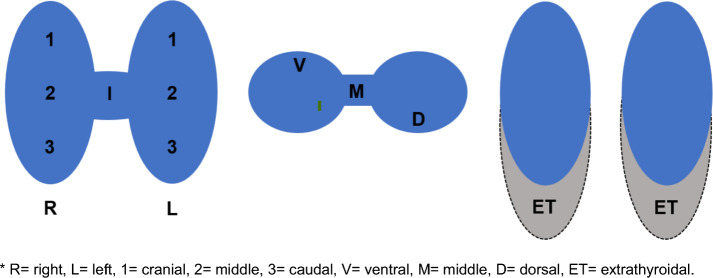
Institutional scheme for localizing thyroid nodule in the thyroid gland

According to study protocol, each reader should select a target nodule and give a localization within thyroid gland using an institutional scheme shown in Fig. 2. Moreover, the target TN was to be evaluated based upon its functionality, assignment to a ACR TI-RADS score, the need for further clarification using SPECT or SPECT/CT, and, finally, whether further imaging had an additive benefit for the final decision regarding the functionality of TN. Since each thyroid examination should include a cine video of thyroid gland including entire nodules, a possible bias of thyroid scan interpretation due to the previous sonographic evaluation or recording was avoided. TNs were interpreted in correlation with sonographic and tomographic findings in line with national and institutional guidelines. Our institutional protocol suggests tomographic or hybrid imaging after planar thyroid scan images, if target TN has a dorsal or atypical location within thyroid gland and is difficult to differentiate based on planar images or sonographically, or there is any sign for thyroid extension into thoracal cavity or tracheal narrowing.

Ultrasound of the thyroid gland was performed using a Hitachi Arietta 850 equipped with a linear probe with a frequency of 5–10 MHz. The institutional ultrasound protocol included regularly volumetry of the thyroid gland (cm^3^), the size of the detected nodules (width, depth, and length, in mm), where most nodules with a size of > 10 mm on one plane were regularly recorded.

Following the reading and data analysing, a consensus panel commissioned by a non-reader board-certified nuclear medicine physician with over 12 years of experience and clinical focus in thyroid diagnostics assessed the cases by determining target TNs and functionality of those TNs after a critical evaluation of provided imaging and clinical information in order to provide a ground truth.

### Statistics

Clinical and demographic characteristics are presented via descriptive statistics. Inter-reader reliability testing for the parameters were performed using Cohen’s for two readers and Fleiss Kappa statistic for more than two readers. The Kappa scale was interpreted as follows: < 0, poor agreement; 0.01–0.20, slight agreement; 0.21–0.40, fair agreement; 0.41–0.60, moderate agreement; 0.61–0.80, substantial agreement; and 0.81–1.00; almost perfect agreement. The association between SPECT(-CT) referral and its additive effect among readers was evaluated by post-hoc analysis using the Chi-Square test. A p value of < 0.05 was considered statistically significant. The descriptive statistical analyses and kappa statistics were performed via Excel Version 2311 (Microsoft® Excel® 2021 MSO), DataTab statistics tool and SigmaPlot 11.0 (Systat Software Inc.) (DATAtab Team 2025). In this study, data cleansing was performed to identify and correct any errors, inconsistencies, or missing values in the dataset, thus improving the overall quality and reliability of the data.

## Results

A total of 61 patients were enrolled in this retrospective study between November 2022 and July 2023, of which were 19 males and 42 females with a mean age of 56 (± 17). As the initial step of reading process, the readers determined a target TN for each patient that was regularly at least 10 mm of size on at least one plane on ultrasonography. Of the target TNs, 33 (54%) were located in the left thyroid lobe, 5 (8%) in the isthmus and the remaining target TNs (38%) in the right lobe. The median target TN size was 20 mm with a range of 10 to 59 mm. According to the consensus panel, there were 2 (3%) TI-RADS level 1 nodules, 8 (13%) TI-RADS level 2 nodules, 44 (72%) TI-RADS level 3 nodules, 6 (10%) TI-RADS level 4 nodules, and 1 (2%) TI-RADS level 5 nodules, as 84% of TN appeared to be assigned as ACR TI-RADS ≥ 3.

For the conduct of further descriptive and inter-reader agreement analyses, we considered only the target lesions that were determined as such by all three readers to ensure the data consistency. The readers determined identical target TNs in 54 patients (89%), whereas the rate of identical target TN determination was 92% among experienced reader. The mean values for thyroid function tests including TSH, free T3 and free T4 were 2.5 (± 0.3), 2.8 (± 0.3) and 14.2 (± 0.4), respectively. According to the consensus reading, the 61% of the target TNs was hypofunctioning, 30% isofunctioning and 9% hyperfunctioning nodules.

### Agreement results

The target nodule match rate among all readers was almost perfect (kappa value: 0.81), while the inter-reader agreement between experienced readers tended to be even better with a kappa value of 0.88. Inter-reader agreement between experienced and inexperienced reader was only slightly lower (kappa value: 0.78). The inter-reader agreement regarding ACR TI-RADS among three readers was moderate with a kappa value of 0.47 (95% CI: 0.40–0.54). However, pairwise comparisons using Cohen’s kappa statistics revealed that the consistency and reliability of ACR TI-RADS categorization has a negative correlation with reader’s experience. For instance, the inter-reader agreement between experienced readers was poor with a kappa value of only 0.27, whereas this was moderate between less experienced readers with a kappa value of 0.44.

Following the determination of target TNs and ACR TI-RADS categorization within thyroid gland, the readers made virtual decisions regarding the referral of patients for tomographic (SPECT) or hybrid imaging (SPECT/CT). The inter-reader agreement regarding patient referral was substantial among all readers with a kappa score of 0.60, while kappa-score was even higher at pairwise comparison of experienced readers (kappa-score: 0.64). The inter-reader agreement with the most experienced and less experienced readers was only fair with a kappa-score of 0.33. Notably, most experienced reader referred further imaging with SPECT(-CT) in 52% of cases, whereas the least experienced only in 25% of the cases.

The inter-reader agreement regarding the target TN functionality among all readers was pretty good with a kappa value of 0.68 (95%CI: 0.59–0.77). Inter-reader agreement between experienced readers was also substantial with a kappa-score of 0.68. Whereas inter-reader agreement assessments involving the least experienced reader revealed slightly lower reliability scores, i.e. a kappa-score of 0.61 between moderate and least experienced readers.

Inter-reader agreement based upon the ground truth results revealed for experienced readers slightly better results than with reader with less experience, i.e. Reader 2, Reader 1 and Reader 3 with kappa-scores of 0.70, 0.69 and 0.65, respectively. Considering the relatively high consistency regarding the functionality of target TNs, we conducted further post-hoc analysis using pairwise Chi-Square tests between each pair of readers in order to determine, whether there was statistical difference in patient referral rate further imaging among readers. This analysis confirmed that there was a significant difference in patient referral pattern between the most experienced and least experienced readers (*p* < 0.01). Table 1 summarizes the results of inter-reader agreement tests.

**Table 1:** Inter-reader reliability among the readers with respect to various clinical and imaging parameters

Feature	Kappa	P–value
Target Nodule Match Rate	0.81	^*^ < 0.001
ACR TI-RADS Categorization	0.47	^*^ < 0.001
Tomography and hybrid imaging referral	0.60	^*^ < 0.001
Target TN functionality	0.68	^*^ < 0.001
Consistency with Ground Truth		
Reader 1	0.69	^*^ < 0.001
Reader 2	0.70	^*^ < 0.001
Reader 3	0.61	^*^ < 0.001

## Discussion

The primary aim of this retrospective study was, to our best knowledge for the first time, to investigate the additive benefit of tomographical/hybrid imaging on thyroid pertechnetate scan with respect to varying reader experience level. Moreover, we conducted inter-reader agreement analyses with respect to target nodule match-rate, and then ACR TI-RADS categorization, functionality and virtual referral rate for further imaging via SPECT(-CT) of those target TNs. The additive benefit of SPECT(-CT) was interpreted based upon comprehensive analysis of the final results and the ground truth.

The main limitations of this study are its retrospective design, lacking histopathological validation of target TNs and selection bias due to inclusion of only patients with ≥ 10 mm-sized TNs who underwent SPECT(-CT) imaging. We sought to investigate the inter-reader agreement regarding multiple aspects of thyroid nodule assessment. Hence, we do not assume any substantial negative effect of the retrospective design on integrity of study conclusions, as prospective design of a study with similar primary aim would not be applicable within routine regular clinical care with a reasonable effort. The patient cohort exhibited, indeed, a certain skewness due to pre-selection of patients. However, as aforementioned, thyroid pertechnetate scan is an integral part of diagnostic work-up of TNs of > 10 mm in regions with goiter due to iodine supply deficiency (Fiore et al. 2014; Holzer and Bartsch 2020). Thus, a study investigating the role of SPECT(-CT) cannot evade this dilemma of patient pre-selection at least in central Europe. In order to avoid bias, the study design ensured a stepwise reporting of findings so that the readers assessed the role of SPECT(-CT) only after the interpretation of ultrasound and thyroid planar imaging. As the focus of this study was the inter-reader agreement, there was no need for any histopathological reference standard. Instead, a reference standard by expert consensus was regarded as sufficient for serving as ground truth in our analyses.

The patient cohort consisted of 61 patients with a mean age of 56 (± 17) years that were predominantly female patients with euthyroid goiter (Table 2). Three readers with different experience levels, i.e. one senior resident with a ≤ 5 -year experience and two experienced board-certified nuclear medicine physicians with > 5-year and 20-year experience, respectively, were supposed to identify the target TN within thyroid gland and assign them to an ACR TI-RADS category based upon the existing sonographic cine video in PACS. The overall agreement was perfect regarding the identification of target TN. However, the overall inter-reader agreement regarding ACR TI-RADS categorization of target TNs was only moderate (k = 0.47; 95% CI: 0.40 − 0.54) which, in addition, exhibited a negative correlation with experience level of readers. This interesting twofold-outcome was in line with previous studies. The meta-analyses by Liu et al. and Li. et al. underline the fact that ACR TI-RADS, despite being one of the most solid TN classifications, exhibits a relatively lower consistency among readers with only moderate pooled inter-reader agreement rates (k = 0.54; 95% CI: 0.49 − 0.58 and k = 0.51, 95% CI: 0.42–0.59, respectively). Furthermore, several studies indicated that readers with lesser experience demonstrate more agreement than the experienced readers. Daniels et al. and Chung et al. stated that this phenomenon would rely on the fact that readers with lesser experience were more prone to follow the criteria for TI-RADS classification strictly than experienced readers, who tend to integrate their clinical experience into their scoring. Even if the inter-reader agreement might be improved after an according reader training, as Du et al. report, ACR TI-RADS classification is associated with an inherent challenge in achieving consistency and reliability among readers (Low et al. 2022; Alfuraih et al. 2024; Li et al. 2022; Liu et al. 2020; Daniels et al. 2021; Chung et al. 2020; Du et al. 2022). Assessing the inherent accuracy of ACR TI-RADS is, however, beyond the scope of our study.

**Table 2:** Patient characteristics

Characteristic	
Gender	
Male	19
Female	42
Age	56 (± 17)
Thyroid gland volume in mL median (range)	20.1 (5–116)
Right lobe volume in mL median (range)	8.4 (1–76)
Left lobe volume in mL median (range)	8.2 (1–73)
Target Nodule size in mm median (range)	20 (10–59)
TSH level	2.5 (± 0.3)
fT3 level	2.8 (± 0.3)
fT4 level	14.2 (± 0.4)
Among identical target TNs	
Number of SPECT Imaging	41
Number of SPECT/CT Imaging	13
Number of hypofunctioning Nodules	33 (61%)
Number of isofunctioning Nodules	16 (30%)
Number of hyperfunctioning Nodules	5 (9%)
[^99m^Tc]O_4_^−^ uptake (TcTU) in % median (range)	1.26 (0.17–8.55)

Overall inter-reader agreement regarding referral rate for further imaging was good (k = 0.60; 95% CI: 0.48 − 0.72), whereas the referral rate for SPECT(-CT) imaging tended to exhibit a positive correlation with increasing experience level. Notably, inter-reader agreement between board-certified nuclear medicine physicians showed the highest value (k = 0.64; 95% CI: 0.47 − 0.81), while inter-reader agreement between the most experienced and the least experienced readers was only fair (k = 0.33; 95% CI: 0.20 − 0.45). In absolute numbers, the most experienced reader made the virtual decision for SPECT(-CT) imaging about twice more than the least experienced reader with a statistical significance (*p* < 0.01; 29 vs. 14 out of 54, respectively). Thus, the positive correlation between referral rate for SPECT(-CT) imaging and reader experience raised question about its effectivity in terms of detection rate of target TN functionality. The overall agreement was also very good regarding target TN functionality (k = 0.68; 95% CI: 0.59 − 0.77), even though experienced readers tended to exhibit higher k-values. When adjusted to the ground truth, individual agreement rates remained at a higher level, i.e. k-values between 0.65 and 0.70.

**Fig. 3:**
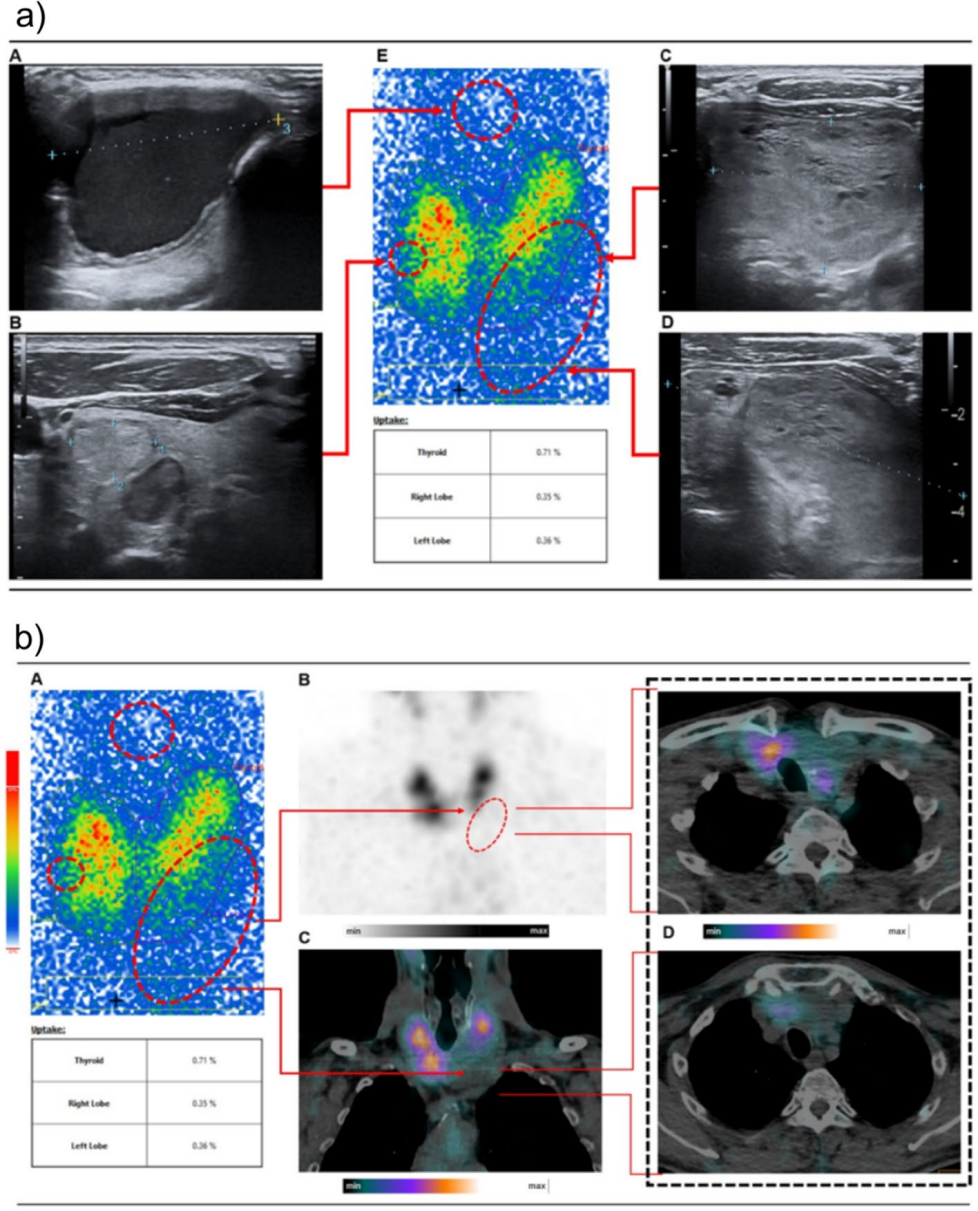
The ultrasound examination revealed solid, clearly margined TN left lower pole of thyroid gland (**B**, **C**, **D**) on a euthyroid goiter. The planar thyroid scan finding was consistent with a hypofunctioning TN (**E**). Furthermore, the ultrasound examination found a cystical non-thyroid structure incidentally. Figure 3B depicts the thyroid enlargement into thoracic cavity resulting in tracheal stenosis and shift to contralateral side in a 59-year-old-male-patient (**A**–**D**) revealed by hybrid imaging

These abovementioned patterns with positive correlation between reader experience and SPECT(-CT) imaging referral rate and its limited potential yield on the final outcome in terms of target TN functionality might seem somewhat intriguing and, thus, require further clarification. We interpret this phenomenon of increased SPECT(-CT) imaging referral with clinical experience as a normal consequence of deep clinical insights of the readers, as with more time in patient care the physicians prefer more nuanced approaches and especially sensitive not to oversee any subtle imaging or clinical signs. This seems to include the anticipation of subtle ultrasound signs for suspected extrathyroidal growth or tracheal narrowing and, beyond that, less experienced readers might operate too task-oriented, i.e. accurate determination of functionality of TN. Even if this more careful patient approach did not deliver a statistically significant outcome in favor of experienced readers in our cohort, this nuanced approach managed to change the therapy course for a few patients and, thus, appeared to be outcome-relevant for a minority of patients. For instance, SPECT/CT imaging revealed a substantial goiter with tracheal stenosis and shift to contralateral side in a 59-year-old-male-patient which represents an indication for surgical treatment (Fig. 3) (Holzer and Bartsch 2020).

## Conclusion

This study provided valuable insights into several aspects of routine diagnostic-work up of incidental thyroid nodules in a tertiary care center. Our analysis indicated that increasing clinical experience of physicians is associated with a higher referral rate of SPECT(-CT) imaging, whereas there is an additive clinical benefit for only in a minority of patients.

In conclusion, SPECT/CT pertechnetate imaging might be more appropriate for high-end tertiary care centers, while its additive benefit in nuclear medicine facilities at primary care level would have negligible benefit for patients in diagnostic work-up of incidental thyroid nodules.

## Supplementary Information

Additional file 1.

## Data Availability

The data used and/or analyzed during the current study are available from the corresponding author on reasonable request.
