# Supplementary material for: Inter-reader agreement and additive benefit of SPECT or SPECT/CT modality with [99mTc]Tc-pertechnetate scintigraphy imaging for the assessment of thyroid nodules in a tertiary care center
Source: EJNMMI Rep. 2025 Aug 18;9(1):29. doi: 10.1186/s41824-025-00264-6 (PMC12358339; doi:10.1186/s41824-025-00264-6)
Supplement: Supplementary file 1 — Additional file 1. [file 41824_2025_264_MOESM1_ESM.pdf]

Supplemental Table

Scanner	Mediso	GE-Healthcare	Siemens	Mediso
**Model**	AnyScan SPECT/CT Flex	DISCOVERY D630	Symbia_T2	Nucline TH-22
**Type**	SPECT/CT	SPECT	SPECT/CT	Gammakamera
**Planar**				
- **Matrix size**		128x128		128x128
- **Zoom factor**		2,57		1,45
- **Acquisition time**		6 min		5 min
- **Energy window**		126,45-154,55 keV		126,45-154,55 keV
- **Pixel size**		1,71		1,41
**SPECT**				
- **Matrix size**	128x128	128x128	128x128	
- **Zoom factor**	2	1	1,23	
- **Number of projections**	120 x 15s	120 x 22s	128 x 20s	
**CT**				
- **Voltage**	120	-	130	
- **mAs**	2	-	30	
- **Collimation**	20mm (single 1,25 mm)	-	5mm (single 2,5 mm)	
- **Pitch**	1	-	1,8	
**Recon**				
- **Algorithm**	OSEM	OSEM	OSEM	
- **settings**	15i 5s 0,9cm Gauss	15i 5s 0,9cm Gauss	16i 4s 0,9cm Gauss	
